# Dr. B. H. Catching

**Published:** 1900-03

**Authors:** 


### Obituary.

### DR. B. H. CATCHING.

It is with great regret that we announce the death of our distinguished *confrere*, Dr. B. H. Catching, of Atlanta, Ga.

It was the writers pleasure to make his personal acquaintance while attending one of the meetings of the Southern Dental Association in Atlanta some twelve years ago, and whilst the distance that separated us prevented any close intimacy, the impression made then, and at several meetings since, leaves a picture of a man whose whole soul was devoted to the welfare of our profession.

On the night before his death he had attended a social entertainment, and arose at the usual hour on the following day, November 23rd, 1899, ate breakfast cheerfully with his family, and was about to start for his office when he was seized with apoplexy and died almost immediately. Whilst we all sincerely mourn the loss of so distinguished a brother, it is impossible not to wish that at the end we may all die as easily.

Dr. Benjamin Holliday Catching was fifty-one years old at the time of his death, having been born at Georgetown, Miss., January 29th, 1848. His great-grandfather was the Honorable Benjamin Catching, a distinguished man of his day, and one of the delegates at the convention which framed the State Constitution.

He studied dentistry first with Dr. J. S. Knapp, of New Orleans, La., and obtained his diploma from the Baltimore Dental College, graduating with honors in 1870, acting as valedictorian of his class. From 1871 to 1881 he practiced at Canton, Miss., removing at the latter date to the City of Atlanta, where he continued in practice until the time of his death.

He has been widely known in the profession from his contributions to literature, but more especially as the editor of the *Southern Dental Journal,* which he conducted for eight years. He resigned this position to carry into effect a cherished project, and began the publication of *Catching's Compendium of Practical Dentistry.* This was a volume published annually for five years, in which the author compiled from the current literature the most useful articles which had been published, classifying them appropriately so that the reader might quickly discover all that was most recent in connection with any given topic. Ill health, largely due to overwork in carrying on this publication, caused him to abandon the enterprise after the fifth volume in 1896, but in 1897 he again undertook literary work, and established *The American Dental Weekly.*

In this venture he had associated with five collaborating editors, and though the little magazine was well received and rapidly achieved popularity, it was abandoned at the end of one year.

The Doctor was once the president of the Southern Dental Association, was a member of the American Dental Association as well as of the more recent National Association. He was prominent in his State Society, and for years served as a member of the Georgia State Board of Dental Examiners.

He leaves a widow, son and three daughters.—*Items of Interest.*

